# Passive hypothermia (≥35 - <36°C) during transport of newborns with hypoxic-ischaemic encephalopathy

**DOI:** 10.1371/journal.pone.0170100

**Published:** 2017-03-09

**Authors:** Aurélie Sellam, Noëlla Lode, Azzedine Ayachi, Gilles Jourdain, Stéphane Dauger, Peter Jones

**Affiliations:** 1 SMUR Pédiatrique, AP-HP, Hôpital Robert Debré, Paris, France; 2 SMUR Pédiatrique, AP-HP, Hôpital André Gregoire, Montreuil-sous-Bois, France; 3 SMUR Pédiatrique, AP-HP, Hôpital Clamart, France; 4 Réanimation Pédiatrique (PICU), Hôpital Robert Debré, Paris, France; 5 Portex Unit, Critical Care Group – Portex Unit, Institute of Child Health, University College London, London, United Kingdom; 6 London School of Hygiene and Tropical Medicine, London, United Kingdom; Centre Hospitalier Universitaire Vaudois, FRANCE

## Abstract

**Background:**

Hypothermia initiated in the first six hours of life in term infants with hypoxic ischemic encephalopathy reduces the risk of death and severe neurological sequelae. Our study's principal objective was to evaluate transport predictors potentially influencing arrival in NICU (Neonatal Intensive Care Unit) at a temperature ≥35-<36°C.

**Methodology/Principal findings:**

A multi-centric, prospective cohort study was conducted during 18 months by the three Neonatal Transport Teams and 13 NICUs. Newborns were selected for inclusion according to biological and clinical criteria before transport using passive hypothermia using a target temperature of ≥35-<36°C. Data on 120 of 126 inclusions were available for analysis. Thirty-three percent of the children arrived in NICU with the target temperature of ≥35-<36°C. The mean temperature for the whole group of infants on arrival in NICU was 35.4°C (34.3–36.5). The median age of all infants on arrival in NICU was 3h03min [2h25min-3h56min]. Three infants arrived in NICU with a temperature of <33°C and eleven with a temperature ≥37°C. Adrenaline during resuscitation was associated with a lower mean temperature on arrival in NICU.

**Conclusions/Significance:**

Our strategy using ≥35-<36°C passive hypothermia combined with short transport times had little effect on temperature after the arrival of Neonatal Transport Team although did reduce numbers of infants arriving in NICU in deep hypothermia. For those infants where hypothermia was discontinued in NICU our strategy facilitated re-warming. Re-adjustment to a lower target temperature to ≥34.5-<35.5°C may reduce the proportion of infants with high/normothermic temperatures.

## Introduction

Hypoxic-ischaemic encephalopathy (HIE) of term new-borns is an important cause of death and neurological disability [1–3]. The frequency of HIE is approximately two to three newborns for every 1000 live births.

Three sequences have been demonstrated to be responsible for the phenomenon in animal studies. There is an initial failure of energy provision before or during birth, a latent phase occurs after reperfusion and finally a biochemical an inflammatory cascade that leads to further energy deprivation and late neuronal death occurring between six and 15h [4]. The neuroprotector effect of hypothermia, as demonstrated in animal models, is to diminish or block late neuronal death.

Reducing cerebral temperature, therapeutic hypothermia, reduces mortality, decreases rates of infant handicap [5–11] and improves cognitive function later in life [2]. Core temperature should be 33.5°C ±0.5°C, or 34.5°C ± 0.5°C for selective head cooling[12]. Therapeutic hypothermia should be initiated as early as possible during the secondary latent phase which corresponds to the first six hours of life [1, 13].

Therapeutic hypothermia loses is effectiveness when introduced late in the secondary latent phase so delay during transport has justified starting hypothermia during transport [14, 15]. The Neonatal Transport Teams (NNT Teams) in the Paris Region currently use a passive cooling method that targets a core temperature of ≥35-<36°C. This approach was approved by the French Neonatal Society (SFN) and Neonatal Study Group, Paris Region (GENIF) to facilitate the acquisition of true hypothermia on arrival in NICU when transport times are short, reduce cases of deep hypothermia (<33°C) whilst enabling rewarming for infants where hypothermia is discontinued on arrival in NCIU.

The principal objective of our study was to evaluate predictors potentially influencing arrival in NICU at a temperature ≥35-<36°C. The secondary objective was to describe treatment assignment to true hypothermia (33–34°C) after arrival in NICU.

## Methods

The study was approved by the local IRB (Comité d’Evaluation d’Ethique en Recherche Biomédicale of the Hôpital Robert Debré) and data recording and processing by the Commission Nationale de l'Informatique et des Libertés. An information letter was provided for parents detailing the inclusion and allowing for the possibility of removal of their child from the study. Consent was not requested. All ethical procedures strictly complied to French law and were approved by the IRB.

The study was a prospective, non-interventional, multi-centric cohort. Recruitment was undertaken between June 2013 and November 2014. Three Neonatal Transport (NNT) Teams (Hôpital Robert Debré [Paris], Hôpital Antoine Béclère [Clamart] and Hôpital André Gregoire [Montreuil]) and 13 NICUs collaborated in the study. The NNT Teams were composed of a Neonatal Transport Consultant, Paediatric Intensive Care Nurse and Ambulance Driver. The decision to start hypothermia was taken by the NNT Paediatrician and/or Maternity Paediatrician sometimes with input from the NICU Paediatrician. All neonates with HIE who were transported by the NNT Teams with therapeutic hypothermia were included if they presented the following criteria: gestational age ≥ 36 completed weeks of amenorrhea, birth weight > 1800g, post-natal age ≤ 6h, and at least one of the following criteria: pH ≤7 and/or base deficit ≥16 mmol/l and/or lactates ≥11 mmol/l during the first hour of life (cord, capillary, venous, arterial), an APGAR ≤ 5 at 10 minutes of life, artificial ventilation at 10 minutes of life and one or more moderate or severe signs of HIE according to the clinical classification of Sarnat (based on level of conscience, posture, tone, spontaneous motor activity and sucking). The criterion for exclusion from the study was core temperature <30°C on arrival of the NNT Team at the maternity. Maternal hyperthermia was defined as a temperature of ≥38°C. Intermediate hypothermia was defined as a temperature of ≥35-<36°C, true hypothermia as a temperature 33–34°C and deep hypothermia <33°C. Maternal, birth and neonatal characteristics were recorded. If hypothermia was instigated prior to the arrival of the NNT Team a target temperature of ≥35-<36°C was used. All temperature control before and during transport was achieved by variation of the ambient temperature.

During transport, passive cooling was performed by turning off the incubator and the removal of clothes and bonnet. The incubator heater could be restarted to raise the temperature. The rectal temperature was continuously monitored (thermal probe Smiths Medical^®^, T400ST09CH connected to a multi-parametric scope Propaq Encore^®^ from Welch Allyn). Recordings of the rectal temperature were made and on arrival of the NPT Team at the maternity and every 10 minutes until arrival in NICU.

Three groups were stratified for analysis according the temperature on arrival in NICU; ≥35-<36°C (reference group), <35°C and ≥36°C.

### Statistical analysis

Qualitative variables were described as percentages. Quantitative variables were described as a mean (standard deviation) or median [inter-quartile range] according to their distribution. Comparative tests were parametric (two-sided, non-paired t-test) or non-parametric (Mann-Whitney) for numeric variables. A Chi^2^ test was used for categorical variables. A regression model was used to study the association between the reference group with factors that potentially could have influenced the temperature on arrival in NICU. Independent numerical, categorical or ordinal variables of the two temperature groups <35°C and ≥36°C were entered separately into the logistic regression model that used the data for the temperature group ≥35-<36°C as a reference. The variables were initially entered in a bi-variate model prior to entry into a backwards multi-variable model for those with a level of significance of <0.20.

A maximum of one variable was entered for every 10 events. Calculations were performed on SPSS software version 22.

## Results

**Fig 1:**
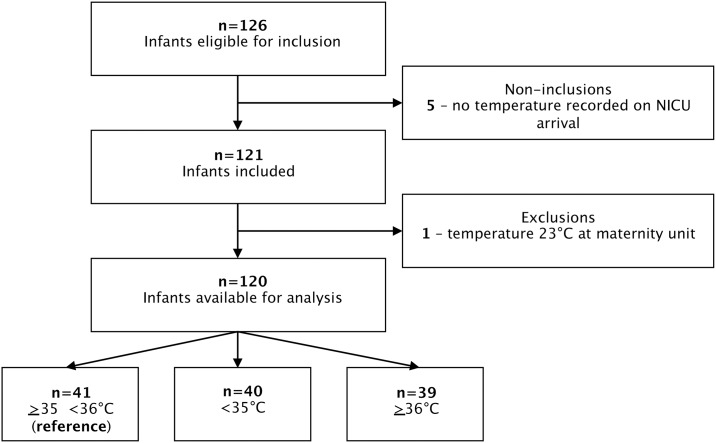
Flow chart illustrating the inclusions, exclusions and repartition of the study group. Three sub-groups with temperatures of ≥35-<36°C (n = 41, reference), <35°C (n = 40), ≥36°C (n = 39) were studied according to the temperature on arrival in Neonatal Intensive Care.

One hundred and twenty-six infants were eligible for inclusion. Five infants were not included and one was excluded for a temperature of 23°C on arrival of the NNT Team; the inclusions and exclusions are shown in Fig 1 and population characteristics are illustrated in Table 1. Forty-four (37%) inclusions were made by the NNT Team of the Hôpital Robert Debré, 41 (34%) by Hôpital André Gregoire and 35 (29%) by Hôpital Antoine Béclère. Forty-five percent were transferred from type 2a maternity units, 18% from type 2b, 30% from type 1 and 8% between type 3. There were no statistical differences in the population characteristics between the three groups (Table 1).

**Table 1:** Population characteristics of the three groups; ≥35-<36°C (reference), <35°C, ≥36°C.

	≥35-<36°C n = 41, reference	<35°C n = 40	p	≥36°C n = 39	p
Mean maternal age (years)	30.6 (26–35.3)	32.4 (26.5–38.3)	0.12	31.0 (26.1–35.9)	0.74
Mean term (weeks)	39.6 (38.2–41.0)	39.1 (37.5–40.7)	0.15	39.7 (38.3–41.1)	0.72
Gravida	2 [1–3]	2 [2–3]	0.16	2 [1–3]	1.0
Parity	2 [1–2]	2 [1–3]	0.30	1 [1–2]	0.34
% Sex (boy)	44 (18/41)	60 (24/40)	0.15	59 (23/39)	0.18
% Foetal rhythm abnormalities	68 (28/41)	82 (32/39)	0.16	71 (27/38)	0.79
% Caesarian	55 (22/40)	59 (22/37)	0.69	47 (18/38)	0.50

The median delay between the call to the NNT Team and arrival at the maternity unit for all infants was 30min [19-41min]. The median age of all infants on arrival in NICU was 3h03min [2h25min-3h56min]. Forty-five percent of the infants had hypothermia initiated by the referring paediatrician prior to the arrival of the NNT Team.

**Fig 2:**
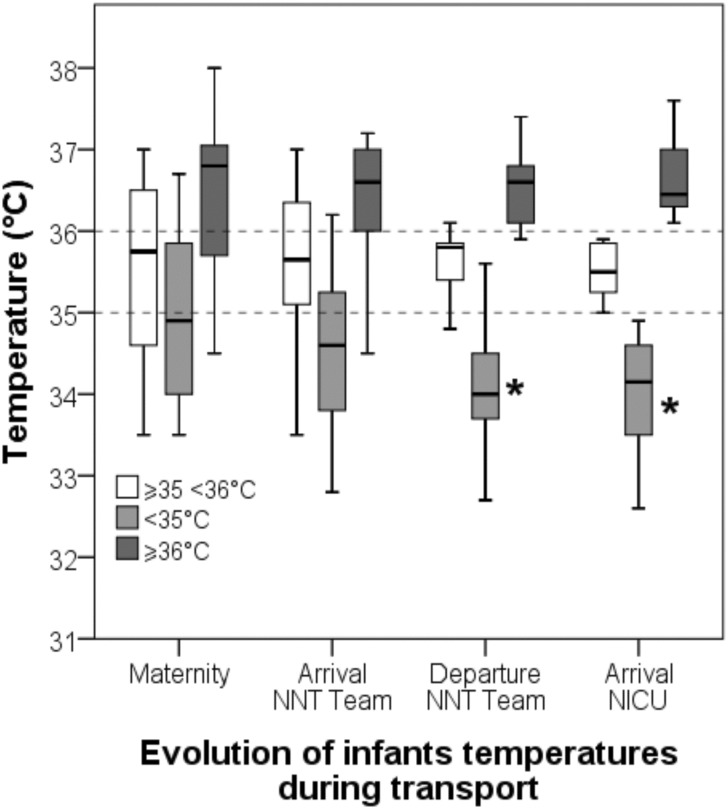
The temperature variation during transport in the three sub-groups according to temperature on arrival in Neonatal Intensive Care (≥35-<36°C [n = 41, reference], <35°C [n = 40°, ≥36°C [n = 39]).

Thirty-three percent of infants arrived in NICU with a core temperature of ≥35-<36°C. The mean temperature recorded in the maternity unit prior to the arrival of the NNT Team was 35.6°C (34.4–36.8°C, n = 72), 35.4°C (34.2–36.6°C, n = 116) on arrival of the NNT, 35.5°C (34.3–37.7°C, n = 113) at the moment of departure to NICU and 35.4°C (34.3–36.5°C, n = 120) on arrival in NICU, see Fig 2.

Nine infants arrived in NICU after six hours of life with a mean temperature of 35.4°C (34.3–36.5). The lowest individual temperatures of children in deep hypothermia on arrival of the NNT were 30.8°C, 31.6°C on departure and 32.6°C on arrival in NICU. The highest temperature on arrival of the NNT was 37.4°C, 37.4°C on departure and 37.6°C on arrival in NICU. Eleven infants (9%) arrived in NICU with a temperature ≥37°C, three of whose mothers had hyperthermia.

Of the 48 infants who received cardio-pulmonary resuscitation (CPR), the 28 received adrenaline and had a significantly lower body temperature (mean temperature 34.6°C [33.7–35.5]) compared to the 20 who did not (mean temperature 36.0°C [34.9–37.1], p<0.0001). The mean temperature of those infants who did not receive CPR was 35.6°C (34.5–36.7) which was not different from those who did receive CPR but not adrenaline (p = 0.39). Adrenaline was identified as an independent predictor of low body temperature in the regression model for the whole group (Table 2). Twelve of 29 (41.4%) infants who received adrenaline were cooled before the arrival of the NNT compared to 38/91 (41.7%) of infants who did not receive adrenaline (p = 0.97).

**Table 2:** Results of the bi-variate regression model comparing variables that cold have had an impact on temperature on arrival in NICU with the three temperature groups; ≥35-<36°C (n = 41, reference), <35°C (n = 40), ≥36°C (n = 39).

Variable	Group	Mean (SD), median [IQR], count (%)	Odds ratio	95% CI	p
Maternal temperature >38.0°C	Reference≥35 - <36°C	5 (2)	Reference		
	<35°C	5 (2)	0.97	0.13–7.3	0.98
	≥36°C	18 (7)	3.8	0.74–19.6	0.11
Mean weight (kg)	Reference≥35 - <36°C	3.23 (2.65–3.81)	Reference		
	<35°C	3.13 (2.51–3.75)	1.3	0.60–2.7	0.48
	≥36°C	3.32 (2.57–4.07)	0.80	0.41–1.59	0.53
Median cord lactates [mmol/litre]	Reference≥35 - <36°C (n = 33)	10.5 [6.9–12.0]	Reference		
	<35°C (n = 32)	10.0 [6.8–14.0]	0.95	0.85–1.07	0.43
	≥36°C (n = 32)	10.5 [6.6–12.0]	1.00	0.89–1.13	0.97
APGAR at 10 minutes	Reference≥35 - <36°C	7 [4–8]	Reference		
	<35°C	6 [4–7]	0.84	0.69–1.03	0.10
	≥36°C	8 [6–8]	1.28	0.99–1.64	0.051
Adrenaline for resuscitation	Reference≥35 - <36°C	22 (9)	Reference		
	<35°C	45 (18)	2.9	1.1–7.7	0.03*
	≥36°C	5 (2)	0.19	0.04–0.95	0.03*

* A significant difference (p<0.05) is shown by an ‘*’.

Higher temperature on arrival in NICU was borderline significantly associated with a higher APGAR score at 10 minutes of life (p = 0.051) and maternal pyrexia was more frequently found in the group arriving with higher temperature (p = 0.11). Infants with a lower APGAR score at 10 minutes generally had a lower temperature on arrival in NICU (p = 0.10). The backwards multi-variable regression model only revealed an association between a higher APGAR at 10 minutes of life and a higher temperature on arrival in NICU (p = 0.04, OR 1.4 [95% CI, 1.01–1.9]).

On arrival in NICU hypothermia was continued in 74/120 (63%) infants. An amplitude-integrated electroencephalogram (aEEG) was performed and the result available for 86 infants. Sixty-two infants had an abnormal aEEG of which therapeutic hypothermia was continued in 90%. For the infants in whom hypothermia was continued on arrival in NICU, the mean temperature was 35.1°C (33.9–36.3) compared to 35.8°C (34.9–36.7) when hypothermia was stopped (p<0.001).

## Discussion

Thirty-three percent of infants arrived in NICU with the target temperature of ≥35-<36°C. The use of adrenaline during resuscitation predicted with a lower arrival temperature. There was a tendency for the infants with higher APGAR scores at 10 minutes or maternal pyrexia to have a higher NICU arrival temperature.

Passive cooling using an intermediate target temperature potentially facilitates acquisition of true hypothermia on arrival in NICU, re-warming if hypothermia is not continued whilst reducing the frequency of deep hypothermia. In the absence of aEEG data at the referring unit there is a potential benefit to this strategy but only when transport times are short and true hypothermia can be obtained within the six hour therapeutic window.

A drawback uncovered by the study was the difficulty in maintaining precise temperature control. Our results are presented looking back from the arrival temperature in NICU because the missing temperature data in the maternity unit would have reduced the size of our cohort. Our study recorded a slight tendency for convergence of the standard deviation towards the mean target temperature (Fig 2). However, the principal limitation of our strategy is the relatively hgh proportion of infants arriving in NICU with high/normothermic temperatures (≥36°C); in particular, nine percent of infants arrived with a core temperature of ≥37°C. A possible future refinement of our strategy would be to lower the target temperature to ≥34.5-<35.5°C. The non-significant association between maternal pyrexia and higher temperature in arrival in NICU may indicate that adjuvant cooling is required from an earlier stage in transport for this group as high temperature in the first three days following HIE has been associated with poor neurological outcome [16].

One of the methodological limitations of our study is the stratification of the results which diluted our statistical power. Another methodological limitation is that whilst our definition of encephalopathy was designed to exclude mild cases by using the Sarnat Score we are unable to differentiate between severe and moderate cases.

Our strategy used was designed to reduce the frequency of deep hypothermia, indeed, only three percent of the group arrived in NICU in deep hypothermia. Previously reported studies of the use of passive hypothermia during transport using a target temperature of 33–34°C have highlighted the risk of deep hypothermia (<33°C).[14, 17–19] Other studies have recorded lowest core temperatures on arrival in NICU of 29.8°C [18, 19], 31.0°C [17] and 29.2°C [14] compared to our lowest recorded temperature of 32.6°C. In one study, as many as one third of infants arrived in NICU with a core temperature of <33°C [23]. Deep hypothermia may induce bradycardia and arrhythmia [20], thrombocytopaenia [6] requiring platelet transfusion [21] although overall haemostasis may not be affected [22].

Our study’s transport times were shorter than those of Fairchild *et al*. [14] who recorded a median time to arrival in NICU of 5.9h (IQR 3.8 to 8.0h) and Kendall *et al*. who recorded a median age at arrival in NICU of 7.24h [23]. In Kendall’s study the use of our intermediate target temperature would have been inappropriate for the majority of those infants transported. However, our results are of value to other NNT Teams, such as Ivaresse et al., who also use passive cooling with a target temperature of 35°C with short transport times (personal communication) [24].

Several authors have acknowledged the difficulty in managing temperature control using passive cooling with or without adjuvants and have recommended servo-controlled cooling. O’Reilly et al. recently looked at three small retrospective cohorts. Of those infants transported using servo-control, 84% arrived in NICU at the target core temperature compared to 47% passive with adjuvants and 20% with passive hypothermia alone [19]. The advantages of servo-controlling are temperature convergence, avoidance of deep hypothermia and precision of control during transport [15, 25]. The disadvantages are cost, technical complexity and the potentially deleterious cooling in infants in whom hypothermia is not pursued in NICU.

Confirmatory evidence to support our finding that APGAR scores influence temperature is available from a 1958 study by Burnard and Cross who established a relationship between 'asphyxia' and decreased rectal temperature in the absence of adrenaline [26]. However, our finding that the administration of adrenaline it is an independent predictor of lower body temperature on arrival in NICU over and above our lower APGAR scores at 10 minute is new. The decision to start hypothermia prior to the arrival of the NNT Team did not influence this difference although another unidentified influence(s) during resuscitation may have been responsible for the association.

Our study revealed 37% of infants in whom hypothermia was discontinued on arrival in NICU. A similar figure to the 30% of 233 infants mentioned in the study of Akula el al. [20] in California who were cooled to 33–34°C inappropriately before and/or during transport for non-HIE pathologies or for mild HIE. Other published articles have not mentioned the discontinuation of hypothermia on arrival in NICU despite very similar criteria for initiating hypothermia [17–19, 23]. This tends to validate our pragmatic strategy of intermediate cooling to avoid the situation described by Hallberg et al. [23] where '…a substantial number of passively cooled… infants arrive with temperatures below the intended range…' and where NICU cooling is discontinued. Others studies of passive cooling in transport have not mentioned discontinuation of cooling on arrival in NICU [14–16, 19, 23], either it did not happen or was not reported. Future publications should mention the proportion of infants in which hypothermia was discontinued on arrival in NICU.

There are three likely reasons for the tendency to interrupt cooling on arrival in NICU. Firstly, although entry criteria for initiation of hypothermia in our study included an 'APGAR ≤ 5 at 10 minutes of life' as one possible criterion, we recorded a median APGAR of 7 at 10 minutes, compared to Kendall *et al*. who recorded a median APGAR of 4.5 at 10 minutes [23] and 4 for Hallberg *et al*. [17] Secondly, there was a high proportion of hypothermia initiated by the Maternity Paediatricians who may have been insufficiently experienced in treatment assignment of hypothermia combined with a reluctance of the NNT Team Paediatrician to discontinue hypothermia in the referring maternity unit. It is unsurprising that Paediatricians sought to instigate hypothermia soon after birth considering the association with improved neurological outcomes [27] and the inevitable delay in achieving true hypothermia that is imposed by transport [28]. Finally, the widespread use of amplitude-integrated electroencephalograms on arrival in NICU had an important role in determining whether hypothermia was continued, or not. Amplitude-integrated EEGs are increasingly being used as a predictor of neurological outcome in HIE [29–31]. The difficulty of treatment assignment using clinical and biological parameters has been recently been highlighted by the absence of reliable evidence-based guidelines for cooling prior to, and during, transport [32].

The infants in our cohort where hypothermia was not continued on arrival in NICU had a significantly higher temperature that those where hypothermia was continued which undoubtedly facilitated both re-warming and attainment of true hypothermia. In future retrieval teams may be appropriate for NNT Teams to have access to aEEG data, or transmit EEG data to level 3 units, to better coordinate treatment assignment at an earlier stage in a similar manner out-of-hospital ECG data and myocardial infarction.

We are following the cohort to determine the association between temperature and neurological outcome.

## Conclusions

Our strategy using ≥35-<36°C passive hypothermia combined with short transport times reduced the risk of the infants arriving in NICU in deep hypothermia. The use a target temperature of ≥35-<36°C would be inappropriate in areas where transport times mean that true hypothermia cannot be achieved within the six hour therapeutic window. For those infants where hypothermia was discontinued on arrival in NICU, our strategy facilitates re-warming. Refinement is needed to increase cooling in cases of maternal pyrexia. The use of adrenaline during resuscitation was an independent predictor of lower body temperature. A re-adjustment of the target temperature to ≥34.5-<35.5°C may reduce the proportion of infants arriving in NICU with high temperatures.

Our methodology may be of particular interest to those in resource limited settings. The effect on neurological outcome is being evaluated.

## Supporting information

S1 Dataset Dataset 1. (XLS)

S2 Dataset Dataset 2. (XLS)
