# Correction: Passive hypothermia (≥35 - <36°C) during transport of newborns with hypoxic-ischaemic encephalopathy

**DOI:** 10.1371/journal.pone.0179068

**Published:** 2017-05-31

**Authors:** Aurélie Sellam, Noëlla Lode, Azzedine Ayachi, Gilles Jourdain, Jean-Louis Chabernaud, Stéphane Dauger, Peter Jones

Dr. Jean-Louis Chabernaud should be included in the author byline. He should be listed as the fifth author and his affiliation is **3** SMUR Pe´diatrique, AP-HP, Hôpital Clamart, France. The contributions of this author are as follows: investigation, contribution of resources, supervision, and funding acquisition.

The correct citation is: Sellam A, Lode N, Ayachi A, Jourdain G, Chabernaud JL, Dauger S et al. (2017) Passive hypothermia (≥35 - <36°C) during transport of newborns with hypoxic-ischaemic encephalopathy. PLoS ONE 12(3): e0170100. https://doi.org/10.1371/journal.pone.0170100.

Additionally, the following information is missing from the Funding Section: The authors would like to thank the GENIF for their generous financial support with the publication of this article.
